# Ten recommendations for reducing the carbon footprint of research computing in human neuroimaging

**DOI:** 10.1162/imag_a_00043

**Published:** 2024-01-29

**Authors:** Nicholas E. Souter, Loïc Lannelongue, Gabrielle Samuel, Chris Racey, Lincoln J. Colling, Nikhil Bhagwat, Raghavendra Selvan, Charlotte L. Rae

**Affiliations:** School of Psychology, University of Sussex, Brighton, United Kingdom; Cambridge Baker Systems Genomics Initiative, Department of Public Health and Primary Care, University of Cambridge, Cambridge, United Kingdom; British Heart Foundation Cardiovascular Epidemiology Unit, Department of Public Health and Primary Care, University of Cambridge, Cambridge, United Kingdom; Victor Phillip Dahdaleh Heart and Lung Research Institute, University of Cambridge, Cambridge, United Kingdom; Health Data Research UK Cambridge, Wellcome Genome Campus and University of Cambridge, Cambridge, United Kingdom; Department of Global Health and Social Medicine, King’s College London, London, United Kingdom; McConnell Brain Imaging Centre, The Neuro (Montreal Neurological Institute - Hospital), McGill University, Montreal, Canada; Department of Computer Science, University of Copenhagen, Copenhagen, Denmark; Department of Neuroscience, University of Copenhagen, Copenhagen, Denmark

**Keywords:** neuroimaging, neuroscience, carbon footprint, computing, sustainability, green

## Abstract

Given that scientific practices contribute to the climate crisis, scientists should reflect on the planetary impact of their work. Research computing can have a substantial carbon footprint in cases where researchers employ computationally expensive processes with large amounts of data. Analysis of human neuroimaging data, such as Magnetic Resonance Imaging brain scans, is one such case. Here, we consider ten ways in which those who conduct human neuroimaging research can reduce the carbon footprint of their research computing, by making adjustments to the ways in which studies are planned, executed, and analysed; as well as where and how data are stored.

## 1 Introduction

We are in the midst of a climate crisis, with exponentially increasing rates of carbon emissions leading to increases in global temperatures. This, in turn, leads to an increased incidence of natural disasters, including floods, fires, and droughts, as well as a loss of biodiversity (IPCC, 2022). Given that the technological path to removing carbon from the atmosphere remains unclear (Carton et al., 2020), and the benefits of carbon offsetting schemes are dubious (Watt, 2021), real-term reductions in emissions will be needed to combat this crisis.

As data-literate individuals with power over the design of research paradigms and the dissemination of knowledge, researchers should reflect on the carbon footprint of their work. One may ask where responsibilities should lie. All actors in the research ecosystem, including governments, institutions, journals, funders, researchers, and data hosts, have a role in incentivising and supporting research practices that promote reductions in carbon emissions (Farley, 2022; Lannelongue et al., 2023; Urai & Kelly, 2023)^1^. While systemic changes are undoubtedly critical, climate conscious researchers should also take initiative through collective action for two important reasons. First, as practising scientists, we have a much deeper understanding of our own research processes than governing bodies, and are therefore well placed to address their impacts. Second, by doing so, we create a social mandate for change with governing bodies. Given there are often barriers to individuals making meaningful change in the face of institutional incentive structures, acting in the domains where we are empowered to act can help in pushing our institutions further. One such domain is research computing.

In many fields, researchers rely heavily on computing. The information communication technology (ICT) sector accounts for an estimated 1.8-3.9% of global CO_2_ emissions, largely accounted for by electricity production (Freitag et al., 2021). Although a small percentage in absolute terms, it is likely to continue growing as we process and store increasing amounts of data. This has become more pertinent in recent years with increasing adoption of large models trained through artificial intelligence (Selvan et al., 2022). Belkhir and Elmeligi (2018) estimated data centre energy use to account for 45% of the greenhouse gas emissions produced within the ICT sector in 2020, up from 33% in 2010. Despite this, the amount of data being collected and processed is relatively neglected in climate policies and initiatives concerning data-driven health research (Samuel & Lucassen, 2022). We therefore urgently need to establish and use best practices for greener computing moving forward. Such initiatives are already being taken in some compute-heavy fields, including bioinformatics (Grealey et al., 2022), machine learning (Selvan et al., 2022), and astronomy (Portegies Zwart, 2020).

Despite initiatives in other disciplines, there has been little attention so far to the computing carbon footprint of human neuroimaging research. This is another compute-heavy field which frequently relies on computationally expensive data processing and analysis. Fortunately, there is scope to reduce this footprint by “computing carefully” (Rae et al., 2022)—reducing a project’s required computing power and, therefore, energy production. Here, we outline several factors that contribute to the energy required for computing in human neuroimaging, and provide ten recommendations for how researchers can reduce these costs (summarised in Box 1). There are a number of other ways in which neuroimaging research contributes to the climate crisis, including through the procurement of specialist equipment, extraction of experimental resources (such as liquid helium for MRI scanning), and frequent flights to international conferences (Aron et al., 2020; Zak et al., 2020). Methods for reducing the carbon footprint of these aspects are beyond the scope of this paper (but see Rae et al., 2022). In the coming years, advances in artificial intelligence may supplement neuroimaging data processing in ways that modulate its carbon footprint—this is also beyond the scope of this present paper.

Box 1. Summary of ten recommendations for reducing the carbon footprint of neuroimaging computing

1. *Preregister a study analysis plan in order to avoid repetitions*
2. *Quantify and report the carbon footprint of your computing using available carbon tracking tools*
3. *Only run the preprocessing and analysis steps that you need*
4. *Run your computing at lower carbon intensity times and in lower carbon intensity locations*
5. *Regularly remove files that you do not need*
6. *Plan where, and for how long, you will store files, aided by research technicians*
7. *Advocate for non-commercial and centralised data storage solutions*
8. *Publicly share sufficient data to ensure it is FAIR (Findable, Accessible, Interoperable, Reusable), but consider the extent of what others will actually need or use*
9. *Make use of existing preprocessed data when possible, instead of acquiring and processing new data*
10. *Discuss the importance of greener computing with other neuroimagers and advocate for systemic change*

## 2 Recommendations

### 2.1 Plan and preregister analysis

Unnecessary repetitions of data analysis represent a waste of energy consumption and should be avoided. Here, we are not referring to replication studies of existing paradigms—these are important in increasing the credibility of science (Open Science Collaboration, 2015). Instead, we are referring to repetitions that occur as the result of unforeseen obstacles. For example, neuroimagers may run analysis for multiple participants, only to discover that results are unusable due to fundamental issues with event timing files (e.g., for fMRI) or missing data. Historically, neuroimagers may have also tweaked analysis pipelines to identify the settings that produced the “best” (i.e., most statistically significant) results. Such repetitions not only contribute to increased carbon emissions, but can also be inconsistent with good research practices. Both issues can be addressed through preregistration of your plan for data collection, preprocessing, and analysis. Preregistration involves uploading a detailed study plan to an online repository (e.g., Open Science Framework (OSF); https://osf.io/registries, AsPredicted; https://aspredicted.org) before data have been collected and/or analysed. Doing so can increase the credibility of your research by clearly delineating between confirmatory and exploratory analyses and providing evidence against suspicions of having “p-hacked” significant results (Gorgolewski & Poldrack, 2016). While writing a preregistration can be initially time consuming, engaging with this process has downstream benefits such as increasing confidence in methods used and improving the efficiency of the analysis stage, thereby reducing the need for unnecessary repetitions and computing. In cases where further exploratory analysis is needed, we recommend that one designs and tests analysis pipelines on a single subject before applying them to the entire sample. This will help with the elimination of code bugs, reducing the amount of unnecessary repetitions and therefore energy use.

***Suggested Action:** Preregister a study analysis plan in order to avoid repetitions*

### 2.2 Track your emissions

In recent years, several tools have been developed to systematically track and quantify carbon emissions associated with computational processes. For example, Green Algorithms (Lannelongue et al., 2021; https://www.green-algorithms.org) is an online calculator that allows users to input parameters for a given job, including runtime, number of cores, and available memory, in order to generate estimates of resulting carbon emissions before a job has started running. This calculator also takes the location of computing into account, given that carbon intensity of energy use will vary by country (see Recommendation 4). It also provides the server-side tool “GA4HPC,” which uses log information to estimate carbon emissions for jobs utilising high performance computing (HPC). Other packages, such as CodeCarbon (Goyal-Kamal et al., 2021; https://codecarbon.io) and Carbontracker (Anthony et al., 2020; https://github.com/lfwa/carbontracker), can be embedded directly into existing tools, allowing researchers to estimate carbon emissions without manually inputting parameters. Again, both packages consider the location of computing, and Carbontracker even makes use of real-time carbon intensity data for a given country, when possible. Recent experiments have shown that these tools provide sensible estimates of energy usage and carbon footprints (Jay et al., 2023).

As of version 22.1.0 (December 12th, 2022), the fMRI preprocessing pipeline fMRIPrep (Esteban et al., 2019) has had CodeCarbon integrated into its code (see https://fmriprep.org/en/stable/changes.html#december-12-2022). Simply by toggling on a “track-carbon” flag and providing a relevant “country-code” (e.g., GBR for United Kingdom) in the command line, fMRIPrep users are provided estimates of carbon emissions for the preprocessing of a given participant. There are strengths and weaknesses to online calculators, server-side tools, and embedded packages, and the ideal solution for a given neuroimager will depend on these factors (Lannelongue & Inouye, 2023a). For example, embedded packages allow automatic collection of computing metrics but are not necessarily compatible with all programming languages, while the reverse is true for online calculators. Whichever approach you use, estimating the carbon footprint of analysis or preprocessing is a good first step to understand the carbon emissions associated with your research computing (Henderson et al., 2020; Lannelongue et al., 2023). Small-scale experimentation, including manipulations of analysis or preprocessing parameters in conjunction with carbon tracking (see Recommendation 3), may further allow researchers to understand which elements of their research computing particularly tax energy usage.

Beyond this, we recommend neuroimagers (and other compute-heavy researchers) provide an “*Environmental impact statement*” in published papers—openly reporting the carbon footprint of their project in kilograms of carbon dioxide equivalent emissions (CO_2_eq; Rae et al., 2022). For particularly compute-heavy projects, this will involve using carbon trackers to provide estimates of the carbon footprint of data processing, whenever available. Box 2 provides an example of the form such a statement could take, based on one pipeline of an ongoing preregistered study focusing on the carbon footprint of fMRI preprocessing (see Recommendation 3).

Box 2. A sample “Environmental IMPACT statement” for a neuroimaging study

“*Preprocessing data for the 257 subjects in the current experiment in fMRIPrep produced an estimated 4.46 kg of carbon dioxide equivalent emissions (CO_2_eq), as determined using an in-house server-side tool (using the same approach as in GA4HPC at https://www.green-algorithms.org). Computing was conducted in the southeast of England, with estimated carbon intensity of 193.38 grams of CO_2_ per kilowatt hour (http://www.carbonfootprint.com*).”

This approach has already been taken by researchers in compute-heavy fields (e.g., Lannelongue & Inouye, 2023b; Xu et al., 2023), and a comprehensive framework for reporting these figures is provided by the Scientific CO_2_nduct initiative (Sweke et al., 2022; https://scientific-conduct.github.io). We encourage researchers to be transparent and pragmatic in reporting this figure. Accurate and representative estimates will allow for synthesis across studies, facilitating a better understanding of which elements of neuroimaging data processing may have a particularly large footprint, and what can be done to reduce this footprint. The adoption of this practice could mirror that of the “*Data availability statement*,” a relatively recent open science initiative that is now culturally accepted within the life sciences, and expected by many journals for the publication of papers.

***Suggested Action:** Quantify and report the carbon footprint of your computing using available carbon tracking tools*

### 2.3 Preprocess conservatively

When working with raw neuroimaging data, preprocessing is a necessary but computationally expensive process. fMRI preprocessing steps include brain extraction, registration, smoothing, and denoising (Caballero-Gaudes & Reynolds, 2017). EEG steps include noise and artefact removal, elimination of bad channels, and re-referencing (Kim, 2018). Following previous lab procedures using existing scripts can be a reliable way to produce good-quality data through a pipeline that runs without error. However, doing so often means that redundant steps are performed which have little or no impact on the final product. From our own experience, existing lab scripts for fMRIPrep have included registration of BOLD data to multiple output spaces and the creation of CIFTI files (storing connectivity data), despite the fact that these files are frequently not used in subsequent analyses. While it may feel useful to store such files “just in case,” it is possible to reduce compute and runtime for your preprocessing by carefully planning which files you will need prior to starting a project. Aspects of analysis may similarly use unnecessary compute. For instance, independent component analysis (ICA) denoising of fMRI data is a computationally lengthy process. While often beneficial in producing higher sensitivity to statistical results, it can operate with varying degrees of success (Scheel et al., 2022). In the absence of good theoretical motivations to conduct steps such as ICA denoising, one should consider which aspects of analysis are necessary.

The scope for meaningful reductions in emissions during job execution will also be impacted by the baseline energy consumption of HPC cluster nodes when idling (not in use). For example, when examining energy costs of running a large language model, Luccioni et al. (2022) found only 54.5% of energy use to be attributable to running code, 13.5% to infrastructure including storage and cooling, and 32% to idling costs needed to keep nodes on regardless of whether code was running on them. Reduction of these costs will likely rely on advances in hardware.

Even within energy costs associated with running a job, it can be challenging for end users to know in advance which preprocessing and analysis steps have meaningful versus negligible effects on compute, as there is a lack of systematic investigation into this question. In the absence of empirical data, researchers may rely on the assumption that individual preprocessing steps that take a particularly long time to complete require particularly intensive compute power. To provide a more systematic estimate, in an ongoing preregistered study (https://osf.io/839pa), we are evaluating the effect of different fMRIPrep parameters on both performance (in analytical sensitivity) and the carbon footprint of preprocessing. Similar investigations using carbon trackers in conjunction with a wider range of packages will allow for a better understanding of how compute power and data quality can be teased apart in order to identify the carbon footprint-optimised set of parameters that balance climate costs and scientific gains. We encourage these future investigations.

***Suggested Action:** Only run the preprocessing and analysis steps that you need*

### 2.4 Time and location matter

**Fig. 1.**
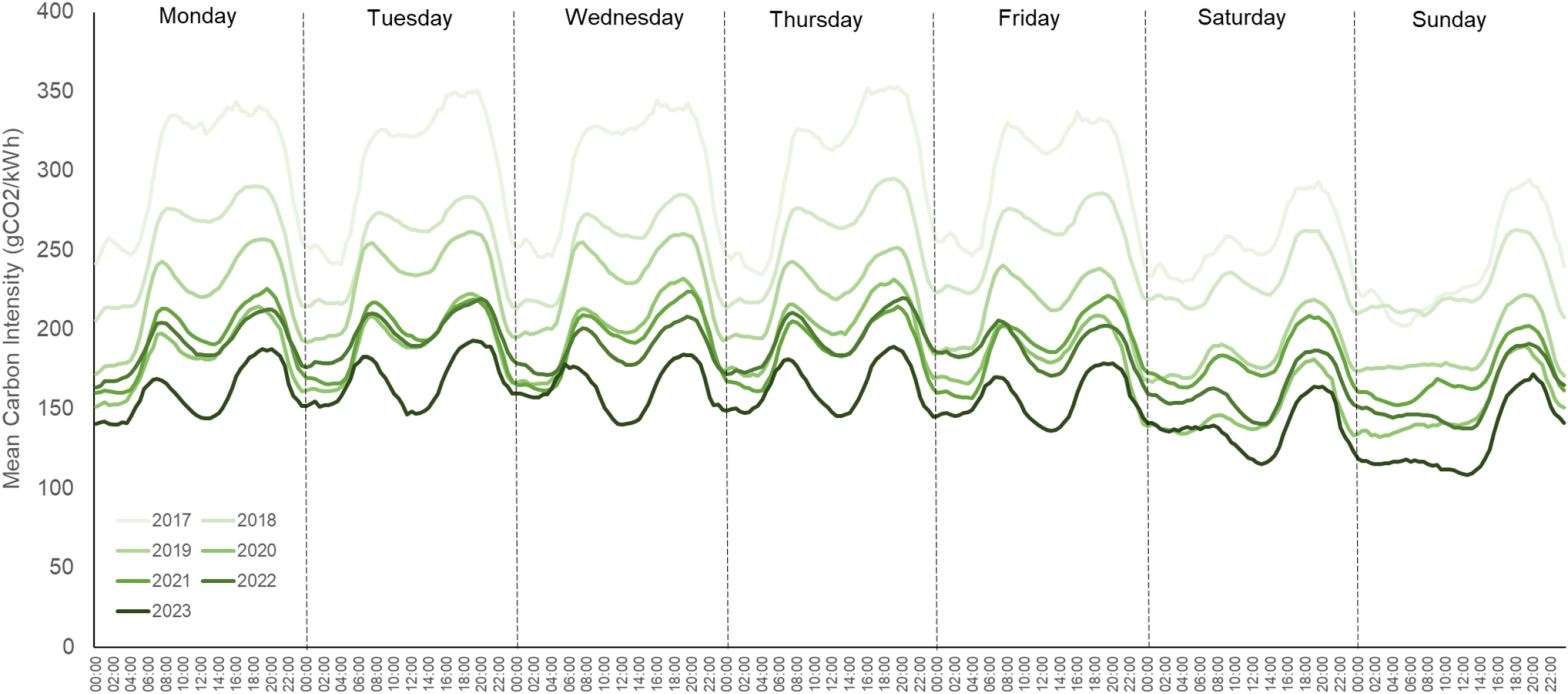
Mean carbon intensity of UK electricity supply for each 30-minute period of the week, split by year from 2017 to 2023. Data taken from the public UK National Grid ESO carbon intensity API (https://carbonintensity.org.uk). Data for 2017 are only available from September 26th to the end of the calendar year. Data for 2023 are presented from the start of the calendar year to October 31st. gCO2/kWh = grams of carbon dioxide per kilowatt hour.

Periods of peak energy use put strain on a national grid’s available renewable energy and increase reliance on carbon-intensive sources in order to meet demand. Data from the UK National Grid ESO carbon intensity API (https://carbonintensity.org.uk), for example, reveal characteristic patterns of carbon intensity, with as much as a 36.8% average decrease from peak to lowest point within a given day of the week (using available data from 2023). Available data from 2017 to 2023 can be seen in Figure 1—with the average carbon intensity presented for each 30-minute period of each day of the week within a given year^2^. During the working week (Monday-Friday), predictable peaks occur at approximately 7:30-8 am and 6:30-7:30 pm—preceding the start and following the end of the working day, when domestic energy use spikes. Carbon intensity is considerably lower overnight and on the weekend. This implies that carbon savings can be made by running analyses at times of lower carbon intensity.

Unfortunately, live carbon intensity data are not publicly available for many countries (see https://app.electricitymaps.com for available sources). However, in cultures following a common 9-5 Monday-Friday working week, peaks and troughs of carbon intensity should approximately resemble those of the UK in Figure 1. When available, live data facilitate the creation of automated job schedulers, which can schedule jobs to run at forecasted periods of low carbon intensity. One recent example is the Climate-Aware Task Scheduler (CATS; https://github.com/GreenScheduler/cats), which can be implemented in the UK for any HPC task. Institutions could take this initiative further by imposing user-specific “carbon budgets” for research computing, in conjunction with task schedulers. While such a move may be controversial among HPC users, this could incentivise researchers to be more mindful in their computing, including the use of scheduling when possible.

The data in Figure 1 should also provide cause for optimism. The transition from 2017 to 2023 reflects an overall reduction in mean carbon intensity in the UK energy mix of 46.4%^3^. Continuing adoption of renewable energy should see this trend continue within the UK. However, given there is a limit to renewable infrastructure that can be created, and that many other aspects of society need to be electrified (e.g., transport), the overall amount of energy available for research computing in a renewably powered world will still be limited.

**Fig. 2.**
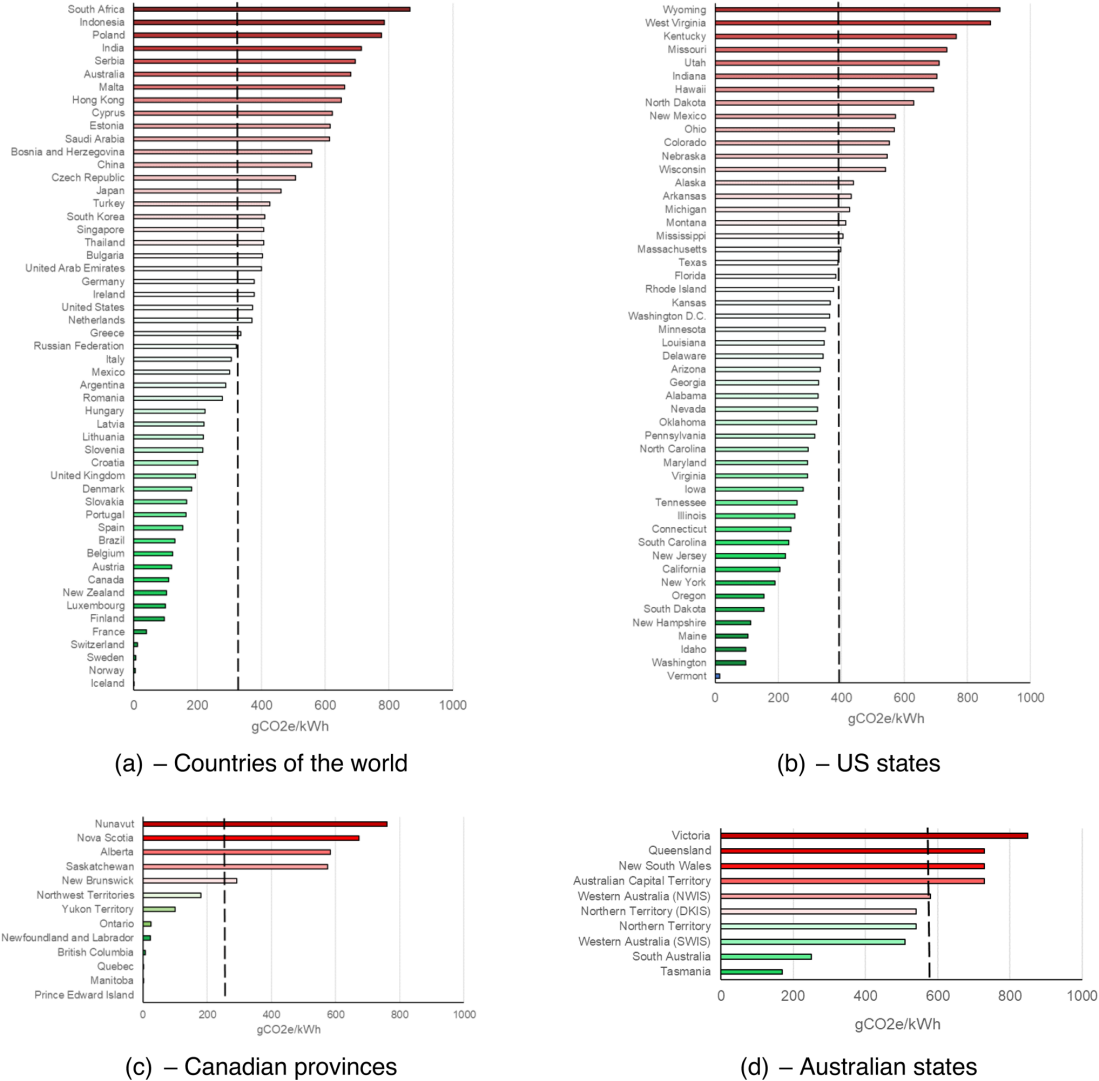
The carbon intensity of energy supply for (a) countries of the world, (b) US states, (c) Canadian provinces, and (d) Australian states. Data taken from the 2022 v1.0 release of Country Specific Electricity Factors (2022) from www.carbonfootprint.com. All available data from this report are plotted. Dotted line reflects the average of countries/states/provinces within each graph. gCO2e/kWh = grams of carbon dioxide equivalent per kilowatt hour. US = United States, DKIS = Darwin Katherine Interconnected System, NWIS = North Western Interconnected System, SWIS = South West Interconnected System.^4^

Carbon intensity also depends on the location in which computing occurs, because energy grids of countries and regions differentially employ carbon-intensive and renewable energy sources. As seen in Figure 2, there is considerable variation in carbon intensity both between and within countries—Iceland’s carbon intensity is 0.01% that of South Africa, due to greater reliance on geothermal-/hydro- and coal-powered energy, respectively. Often, it will be difficult for neuroimagers to adjust the location in which computing is done, as researchers may be tied to the physical location of institutional servers. This becomes more tractable when considering cloud computing services, which may house servers in more or less carbon-intensive areas. When storing and sharing data, researchers may choose to prioritise data repositories supported by servers in areas with low carbon intensity. However, researchers should remain cognisant of the implications of their choices in terms of, for example, any socio-political issues associated with where data centres are constructed, and any ramifications on local areas and/or communities. Well thought-through and equitable international inter-institutional collaborations may be critical in providing researchers in low- and middle-income countries with access to low carbon intensity computing opportunities (Lannelongue et al., 2023). Overall, by scheduling preprocessing or analysis to run at periods or in locations of low carbon intensity, you could emit considerably less carbon while using the same amount of energy.

***Suggested Action:** Run your computing at lower carbon intensity times and in lower carbon intensity locations*

### 2.5 Tidy up “junk” files

Energy is required not only to process data, but also to store it. Increasing the amount of data stored on a server can impact workload, by providing a larger amount of material to backup, for instance. The mere storage of data also incurs energy consumption due to the requirement for powering hard drives and air conditioning in server rooms. The more we store, the greater the energy consumption. Additionally, as institutions run out of space to store data, it becomes necessary to acquire additional hardware. Even before servers are in use, the production of computing hardware contributes a substantial portion of the carbon impact for this sector, 15-40% for data centre servers^5^, and 70-90% for consumer devices (Clément et al., 2020). Overall, 10 kg of CO_2_-equivalent is the order of magnitude of the carbon footprint of each terabyte of data stored on a hard drive (Lannelongue et al., 2021).

**Fig. 3.**
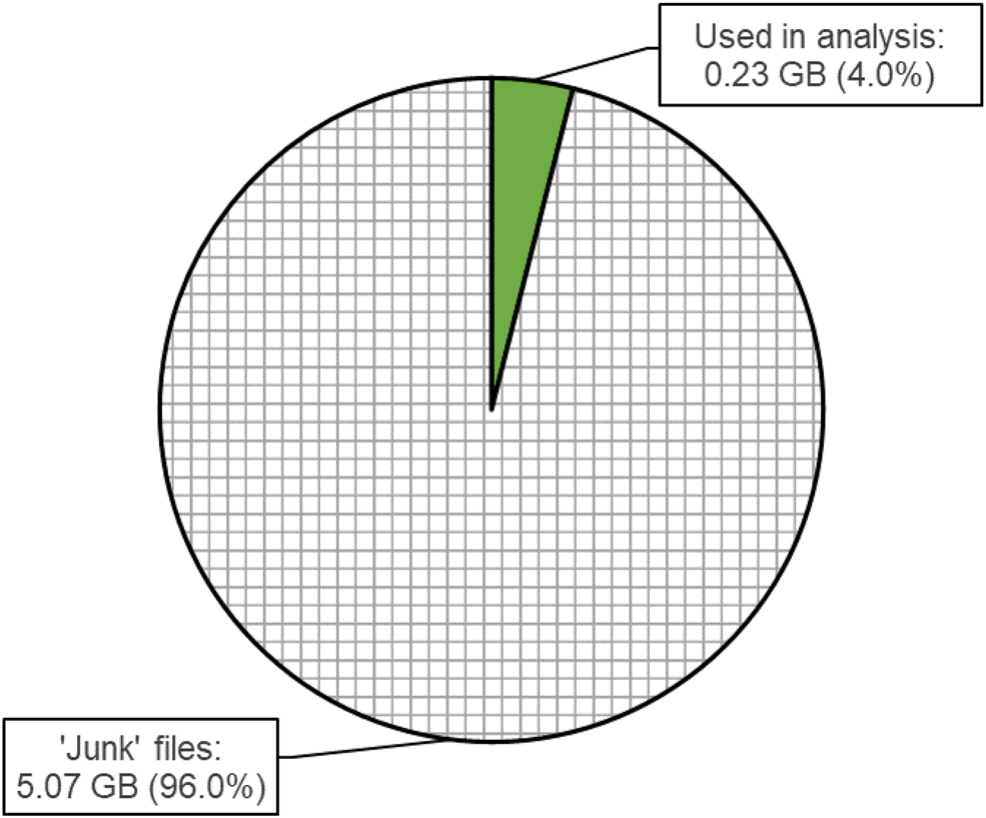
The mean percentage of total data generated by fMRIPrep that is actively used in data analysis (solid green) versus files that can be safely deleted after the completion of preprocessing (chequered). Data correspond to preprocessing of one run of a stop signal task for 257 subjects (Bilder et al., 2020; https://openneuro.org/datasets/ds000030/versions/1.0.0), and includes working directory files, derivatives, logs, and figures. GB = gigabytes.

It is common for neuroimaging pipelines to produce large amounts of intermediary files that will never be used by the researcher. This includes files generated both in working directories and for the final output. For the aforementioned preregistered study (https://osf.io/839pa), we have been processing data for 257 subjects in fMRIPrep. For a single pipeline, fMRIPrep generated a total average of 5.55 GB per subject (across output files, working directories, and logs). Only 0.23 GB, 4.0% of the total size, corresponded to files intended for use in subsequent statistical analysis (see Fig. 3). To address this unnecessary output, we provide an open source tool, fMRIPrepCleanup, available for download on GitHub (https://github.com/NickESouter/fMRIPrepCleanup), designed to delete unnecessary fMRIPrep files within a given directory. Using such automated scripts to cleanup junk files can place less stress on existing storage infrastructures and reduce the need for additional server purchases and manufacturing. However, extreme care should be taken when writing and executing such scripts, including the one linked here. They will need to be customised based on the research needs of the user and the output file structure they have created. If the overarching directory and file paths to be saved are not correctly specified, you risk irrevocably deleting important data. We recommend executing such a script on a copy of one participant’s dataset first, and using the Brain Imaging Data Structure (BIDS) file organizational structure (Gorgolewski et al., 2016) to make it easier to index files that are to be kept and deleted as appropriate. It is typically more energy-intensive to regenerate files than it is to store them. As such, files should only be deleted following an evaluation of exactly what will need to be retained, in order to avoid unnecessary repetition.

***Suggested Action:** Regularly remove files that you do not need*

### 2.6 Plan your long-term storage

As well as making efforts to delete unneeded files, researchers should consider the method and duration of storage of files that do have value. As covered in Recommendation 5, the long-term storage of files has a carbon footprint. Researchers in computationally expensive fields such as neuroimaging can explicitly consider the carbon footprint implications of data storage in data management plans prior to beginning a study, as outlined by the Digital Humanities Climate Coalition (DHCC Information, Measurement and Practice Action Group, 2022; https://sas-dhrh.github.io/dhcc-toolkit). Many institutions have policies requiring researchers to retain data on local storage for a minimum time period; these are sometimes specific in scope (e.g., 10 years) or are sometimes vague and unspecified (Briney et al., 2015; e.g., that data should be retained for an “appropriate” period of time, as judged by the researcher). It is unclear what typically happens to data after such a retention period. Although there may be an implicit assumption that researchers delete data after this period, they may not do so without explicit encouragement. This can contribute to the accumulation of “dark data” that is poorly indexed or simply unneeded, and therefore becomes functionally invisible and unused while taking up space (Schembera & Durán, 2020). Here, institutional research technicians or administrators could play a vital role in prompting researchers to regularly remove files that are no longer needed or within the retention period. Having recommended options for data at the end of a project’s life cycle would help avoid the accumulation of dark data. For neuroimagers concerned about permanently losing access to data, transitions from digital disk storage to long-term tape storage may provide substantial savings in both storage costs and carbon emissions (Johns, 2020), as tape storage does not require energy to air condition servers or cover baseload. Researchers could consider utilising offline or solid storage for their data unless they have specific reasons not to do so.

***Suggested Action:** Plan where, and for how long, you will store files, aided by research technicians*

### 2.7 Push for publicly owned centralised data storage

Centralised data storage avoids the duplication of datasets in each research group and benefits the research community as well as the environment. General open science repositories, such as the OSF (https://osf.io), and neuroimaging-specific platforms, such as Neurovault (https://neurovault.org) and OpenNeuro (https://openneuro.org), allow neuroimagers to provide public access to raw and processed neuroimaging data. This practice has helped make neuroimaging research more credible, reproducible, and accessible (Gorgolewski & Poldrack, 2016). Although larger data centres tend to be more energy efficient than decentralised small data storage infrastructures (Masanet et al., 2020), it is important to assess the carbon footprint of these facilities when building such centralised storage resources. Moreover, these repositories rely on commercial cloud computing services. For instance, data for OSF are hosted by Google, and data for OpenNeuro are hosted by Amazon Web Services. Some researchers may perceive ethical issues with entrusting public medical data to such commercial platforms—including issues surrounding privacy and data security, and ownership and control (Chiruvella & Guddati, 2021). BigTech is also associated with producing and amplifying a range of social injustices and inequalities (Couldry & Mejias, 2018). Besides, claims made by large cloud providers around renewable energy and sustainability can be difficult to verify—transparency on this matter is not always evident.

Such concerns may be addressed through the use of publicly owned, non-commercial “trusted research environments” (TRE), which allow secure access to large amounts of medical data (Graham et al., 2022), and are run for, and by, the research community. When operating at a large scale, such services can also have power use effectiveness comparable to that of commercial services. The European High Performance Computing Joint Undertaking (EuroHPC JU) provides a promising example of a non-commercial approach (https://eurohpc-ju.europa.eu). For example, one EuroHPC JU initiative, LUMI in Finland, reportedly ranks as one of the most energy-efficient supercomputers in the world while also being fully powered by renewable hydroelectric energy^6^. When considering data storage, non-commercial solutions such as this may provide an optimal balance between computing power, energy efficiency, and transparency. While the power of individual neuroimagers in this sense is limited, researchers can help here by advocating for and supporting the use of non-commercial computing initiatives and TREs.

***Suggested Action:** Advocate for non-commercial and centralised data storage solutions*

### 2.8 Reflect on what needs to be shared

In a recent survey of neuroimaging researchers, 54% indicated that they were likely to share all raw imaging data in online repositories for their next project (Paret et al., 2022). This demonstrates an impressive commitment from much of the neuroimaging community to transparent and accountable science. When doing so, researchers should ensure that the data they share are FAIR—findable, accessible, interoperable, and reusable (Wilkinson et al., 2016). These principles are often not adhered to (Crüwell et al., 2023), and sharing of unFAIR data may in some cases be worse than sharing nothing. Access to all data for a project can significantly enhance the utility of a dataset. However, as the demands on the ICT sector continue to grow, data centres will require more energy and space to operate (Freitag et al., 2021; although see Masanet et al., 2020 for an opposing view). This will also manifest as increased costs to data sharing platforms^7^. Even technological improvements in cloud computing may elicit a rebound effect, whereby increasingly efficient data storage actually leads to net increases in demand and therefore storage space and energy used (Widdicks et al., 2023). For their part, researchers should consider which aspects of their data will realistically be necessary or helpful to share. In most cases, it may be sufficient to upload preprocessed data only—enough to replicate analysis and test novel hypotheses, while placing minimal strain on cloud computing. Additionally, sharing too much can make it harder for users to navigate and correctly use datasets. We are not advocating against the sharing of data. However, exponential increases in the amount of existing data may make it necessary to ask difficult questions. Ultimately, it may be wise for researchers, institutions, or data-sharing platforms to place expiration dates on datasets, with removal after a set period. For now, researchers can exercise their own best judgement on the balance between the usefulness and size of their public data. Neuroimagers may benefit from the publication of a consensus paper, providing guidelines for sharing imaging data with sufficient rigour while also considering greener computing.

***Suggested Action:** Publicly share sufficient data to ensure it is FAIR (Findable, Accessible, Interoperable, Reusable), but consider the extent of what others will actually need or use*

### 2.9 Use existing data

In neuroimaging investigations, the default approach is to design a novel paradigm and collect raw data from a novel sample. Making use of pre-existing, and often preprocessed data which are already suitable for statistical analysis, allows one to save time and resources, and importantly to avoid the energy use associated with processing novel data (a computationally expensive process, see Recommendation 3; Rae et al., 2022). This will only apply when the data necessary to answer a research question already exist, and are publicly available (see Recommendation 8). Large public datasets for research use include the Human Connectome Project (https://www.humanconnectome.org; Van Essen et al., 2013), which incorporates multimodal datasets across young adult, developmental, ageing, and clinical samples. These contain task-based fMRI, resting-state fMRI, MEG, and PET data. Similarly, UK Biobank is a mass-scale study comprising diverse phenotypic and genotypic data, including structural, diffusion, and functional MRI (https://www.ukbiobank.ac.uk; Miller et al., 2016). As of October 2022, over 60,000 volunteers have been imaged, with a target of 100,000 individuals in the final sample^8^. These data are used to generate over 4,000 imaging-derived phenotypes—metrics such as structure volume and connectivity that can be used as predictors of disease risk factor (Alfaro-Almagro et al., 2018). Note that this platform does not offer free access to its data, meaning it is not an entirely accessible public resource. Alternatively, the platform OpenNeuro provides free access to over 800 public datasets spanning MRI, fMRI, PET, MEG, EEG, and iEEG data (https://openneuro.org). Other open data repositories are listed in Table 1^9^. When possible, the re-use of existing data provides a good example of how open science practices can intersect with opportunities to reduce one’s personal compute emissions.

**Table 1.** Overview of open access neuroimaging projects/data repositories, with available links, modalities, countries of origin, dataset sizes, and brief descriptions.

Project	Link	Imaging modalities	Based in	Dataset size (participants)	Description
A large and rich EEG dataset for modeling human visual object recognition	https://figshare.com/articles/dataset/A_large_and_rich_EEG_dataset_for_modeling_human_visual_object_recognition/18470912	EEG	Germany	10	Contains EEG data for responses to images of objects on a natural background. Ten participants each with a large number of trials.
Adolescent Brain Cognitive Development (ABCD) study	https://abcdstudy.org	MRI, fMRI, dMRI	US	11,880	A large long-term study of brain development and child health.
Alzheimer’s Disease Neuroimaging Initiative (ADNI)	https://adni.loni.usc.edu	MRI, PET	US	800 (ADNI 1)507 (ADNI 2)	A longitudinal multicenter study designed to develop biomarkers for the early detection of Alzheimer’s disease.
Amsterdam Open MRI Collection (AOMIC)	https://openneuro.org/datasets/ds002785/versions/2.0.0	MRI, fMRI, dMRI	Netherlands	216	Multiple large datasets containing data for various task-based fMRI paradigms, psychometrics, and demographics.
Autism Brain Imaging Data Exchange (ABIDE)	https://fcon_1000.projects.nitrc.org/indi/abide	MRI, fMRI, DTI	US/Europe	1,112 (ABIDE 1)1,000+ (ABIDE 2)	Aggregates data from institutions around the world to further our understanding of the neural bases of autism.
Cambridge Centre for Ageing and Neuroscience (Cam-CAN)	https://www.cam-can.org	MRI, DTI, DKI, MEG, fMRI	UK	623-653 (varies by imaging modality)	A large collaborative project, focused on how individuals can retain cognitive abilities into old age.
Enhanced Nathan Kline Institute - Rockland Sample (NKI-RS)	https://fcon_1000.projects.nitrc.org/indi/enhanced	MRI, fMRI	US	1,000+	A large community sample of participants across the lifespan, contains diverse data types.
ERP CORE	https://erpinfo.org/erp-core	EEG	US	40	Contains event-related potential data for six paradigms relating to different components.
Human Connectome Project (HCP)	https://www.humanconnectome.org	MRI, fMRI, MEG, PET	US	1,200 (Young Adult) 1,200 (Aging) 1,350 (Development) 500 (Lifespan Baby) 1,500 (Lifespan Developing) Various clinical patient datasets	Contains multiple large datasets spanning different age groups across diverse tasks.
Imaging and Data Archive (IDA)	https://ida.loni.usc.edu	MRI, CT, SPECT, PET, EEG	US	Signposts 150 studies; 95,100 participants	A resource for archiving and signposting neuroscience data repositories, including some of those listed here.
International Neuroimaging Data-Sharing Initiative (INDI)	http://fcon_1000.projects.nitrc.org	MRI, fMRI	US	1,200+	Includes a public release of resting-state fMRI datasets from 33 sites.
MEG UK	https://meguk.ac.uk/database	MEG, MRI	UK	~500 (prospective)	A partnership between eight UK labs, adding towards a single shared repository of MEG data.
Mother of Unification Studies (MOUS)	https://data.donders.ru.nl/collections/di/dccn/DSC_3011020.09_236?0	MRI, fMRI, MEG	Netherlands	204	Multimodal data, includes a language task and resting-state data. In BIDS format.
Multisubject, multimodal face processing	https://openneuro.org/datasets/ds000117/versions/1.0.3	MRI, fMRI, MEG, EEG	UK	16	A multimodal dataset focused on face processing conducted over two sessions. In BIDS format.
Natural Scenes Dataset (NSD)	http://naturalscenesdataset.org	Ultra high-field 7 T MRI, fMRI	US	8	Data for eight participants, viewing thousands of colour natural scenes over 30-40 scans.
Neurosynth	https://neurosynth.org	fMRI	US	Aggregates existing results	Allows for the automatic synthesis of existing fMRI data for studies focusing on a given topic or function. Produces statistical maps of activation.
Neurovault	https://neurovault.org	MRI, fMRI, PET	US	Many separate datasets	A public repository of unthresholded statistical maps derived from neuroimaging studies.
Open Access Series of Imaging Studies (OASIS)	https://www.oasis-brains.org	MRI, fMRI, DTI, PET	US	416 (OASIS-1) 150 (OASIS-2) 1,379 (OASIS-3) 451 (OASIS-3_TAU) 663 (OASIS-4)	A project aimed at making neuroimaging datasets freely available for download. Contains five distinct datasets largely focused on ageing and dementia.
OpenNeuro	https://openneuro.org	MRI, fMRI, PET, MEG, EEG, iEEG	US	800+ datasets	A free platform that provides access to over 800 public datasets, all BIDS-compliant. Formerly “OpenfMRI”.
Release of cognitive and multimodal MRI data including real-world tasks and hippocampal subfield segmentations	https://datadryad.org/stash/dataset/doi:10.5061/dryad.2v6wwpzt3	MRI, dMRI, fMRI,	UK	217	Extensive cognitive assessment and neuroimaging data for a neurologically healthy sample. Aimed at understanding neural bases of individual difference, particularly in the hippocampus.
StudyForrest	http://www.studyforrest.org	MRI, fMRI, dMRI	Germany	20	A project centering around the movie Forrest Gump, providing highly reproducible scanning of rich contexts.
UK Biobank	https://www.ukbiobank.ac.uk	MRI, fMRI, dMRI, IDP	UK	60,000+	A biomedical database containing data from UK participants. The world’s largest imaging study.

Note: This list is not exhaustive. MRI = magnetic resonance imaging, fMRI = functional MRI, dMRI = diffusion MRI, PET = positron emission tomography, MEG = magnetoencephalography, IDP = imaging-derived phenotypes, EEG = electroencephalography, iEEG = intracranial EEG, CT = computerised tomography, SPECT = single-photon emission CT, DTI = diffusion tensor imaging, DKI = diffusion kurtosis imaging, US = United States, UK = United Kingdom, BIDS = brain imaging data structure. Other lists of neuroimaging databases are available (e.g., https://en.wikipedia.org/wiki/List_of_neuroscience_databases; https://imaging.mrc-cbu.cam.ac.uk/methods/OpenDatasets; https://sccn.ucsd.edu/~arno/fam2data/publicly_available_EEG_data.html)

***Suggested Action**: Make use of existing preprocessed data when possible, instead of acquiring and processing new data*

### 2.10 Talk about greener computing

When we talk to neuroimaging colleagues about greener computing, many say they have not previously considered the carbon emissions associated with this aspect of the research process. This carbon footprint is perhaps challenging to intuitively conceptualise, compared to more visible sources of carbon emissions such as aviation. However, by reflecting on the above recommendations and actively discussing them with your neuroimaging community, you can raise awareness about this issue. In recent years, we have been inspired by increasing engagement on environmental sustainability issues within the neuroscience community, from conference attendance and participation in green neuroscience- and computing-themed sessions (e.g., British Neuroscience Association 2021, “*Environmental impacts of computing in health & life sciences research”* workshop 2023), to environmental chapters within neuroscience societies (e.g., the Sustainability and Environment Action Special Interest group in the Organization for Human Brain Mapping). As with recent drives for open science practices (Gorgolewski & Poldrack, 2016) and equitable publication costs (Sanderson, 2023) within this community, there is an appetite amongst neuroimagers to be more environmentally conscious in their work. Neuroimagers can—and we believe should—exercise advocacy in their own work, challenging established norms that exacerbate the footprint of the field.

Individual researchers may feel that their actions have a negligible impact on net emissions. However, fostering a culture of thinking seriously about these issues will contribute to the implementation of ideas into standard practice, as we have seen with the open neuroimaging movement. Systemic change from governments, institutions, and funders will be critical in facilitating this change. Alongside this, however, researchers should take small steps in their own work when possible. This can include the implementation of novel practices, such as “*Environmental impact statements*” (see Recommendation 2), HPC task scheduling, and carbon budgets (see Recommendation 4). We call on all neuroimagers to actively consider the carbon footprint of their work, especially that derived from computing, where there are already meaningful steps that can be taken.

***Suggested Action:** Discuss the importance of greener computing with other neuroimagers and advocate for systemic change*

## 3 Conclusion

We have discussed 10 ways in which neuroimagers can reduce the carbon footprint of their research computing. As data-literate individuals in positions of power, with the ability to influence the use of research funding, scientists have an obligation to consider the impact of their work. When working with large amounts of data, neuroimagers should reflect on how efficiently these data are processed, stored, and shared. We hope that the recommendations outlined here will help to foster a culture of addressing the environmental impacts of neuroimaging research computing.

## Supplementary Material

Supplementary Material

## Data Availability

Raw data for Figure 1 and Figure 2 are available through the sources cited in the respective figure headers. All processed data used to generate figures for this paper, and the code used to process them, are publicly available on the Open Science Framework (Souter, 2023; https://osf.io/kq9ue).

## Footnotes

1 Indeed, recent reports from research funders demonstrate interest in this topic, Wellcome; https://wellcome.org/reports/advancing-environmentally-sustainable-health-research, UKRI; https://zenodo.org/record/8199984

2 Changes in carbon intensity by month are not entirely consistent across years, see Supplementary Figure 1.

3 At the time of writing, data were not available for all of 2017 or 2023. The equivalent reduction from 2018 to 2022 is 26.3%.

4 Note that exact estimates of carbon intensity vary by source; there is disparity between numbers presented here and those provided by Our World in Data (https://ourworldindata.org/grapher/carbon-intensity-electricity), although the rank order of countries by intensity across sources is comparable.

5
https://www.dell.com/en-uk/dt/corporate/social-impact/advancing-sustainability/climate-action/product-carbon-footprints.htm#scroll=off&tab0=3

6
https://eurohpc-ju.europa.eu/eurohpc-supercomputers-are-still-among-fastest-and-greenest-2023-05-22_en

7
https://www.cos.io/blog/shared-investment-in-osf-sustainability

8
https://www.ukbiobank.ac.uk/learn-more-about-uk-biobank/news/world-s-most-ambitious-imaging-study-scans-60-000th-participant

9 As presented in this table, there are issues here with the diversity of data— all projects found are based in the US or Europe. All sources appear free to access, with the exception of UK Biobank.
