# Supplementary material for: Ten recommendations for reducing the carbon footprint of research computing in human neuroimaging
Source: Imaging Neurosci (Camb). 2024 Jan 29;1:imag-1-00043. doi: 10.1162/imag_a_00043 (PMC12007543; doi:10.1162/imag_a_00043)
Supplement: Supplementary Material [file imag_a_00043-supp.pdf]

Ten recommendations for reducing the carbon footprint of research computing in human neuroimaging

Supplementary Materials

Nicholas E. Souter [a], Loïc Lannelongue [b, c, d, e], Gabrielle Samuel [f], Chris Racey [a], Lincoln J. Colling [a], Nikhil Bhagwat [g], Raghavendra Selvan [h, i], Charlotte L. Rae [a]

[a] School of Psychology, University of Sussex, Brighton, United Kingdom

[b] Cambridge Baker Systems Genomics Initiative, Department of Public Health and Primary Care, University of Cambridge, Cambridge, United Kingdom

[c] British Heart Foundation Cardiovascular Epidemiology Unit, Department of Public Health and Primary Care, University of Cambridge, Cambridge, United Kingdom

[d] Victor Phillip Dahdaleh Heart and Lung Research Institute, University of Cambridge, Cambridge, United Kingdom

[e] Health Data Research UK Cambridge, Wellcome Genome Campus and University of Cambridge, Cambridge, United Kingdom

[f] Department of Global Health and Social Medicine, King's College London, London, United Kingdom

[g] McConnell Brain Imaging Centre, The Neuro (Montreal Neurological Institute - Hospital), McGill University; Montreal, Quebec, Canada

[h] Department of Computer Science, University of Copenhagen, 2100, Copenhagen, Denmark

[i] Department of Neuroscience, University of Copenhagen, 2200, Copenhagen, Denmark

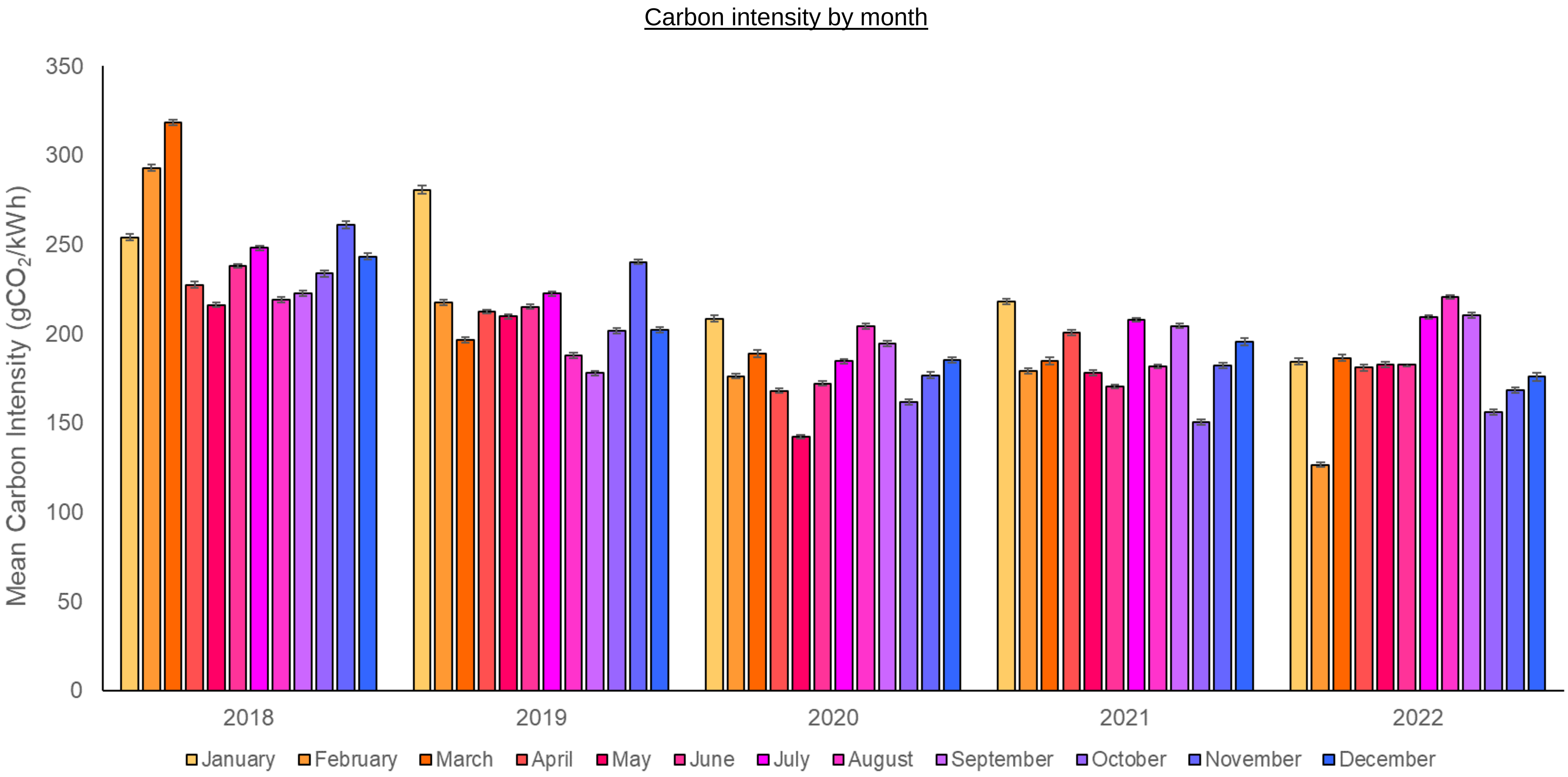

Supplementary Figure 1. Mean carbon intensity split by month from 2018-2022. Data taken from the public UK National Grid ESO carbon intensity API (https://carbonintensity.org.uk). Data not presented for 2017 or 2023 given that data for the whole year is not available in these cases. gCO_2/kWh = grams of carbon dioxide per kilowatt hour. Error bars reflect one standard error of the mean
